# Battle for Climate and Scarcity Rents: Beyond the Linear-Quadratic Case

**DOI:** 10.1007/s13235-015-0154-2

**Published:** 2015-05-28

**Authors:** Mark Kagan, Frederick van der Ploeg, Cees Withagen

**Affiliations:** VU University Amsterdam, De Boelelaan 1105, 1081 HV Amsterdam, The Netherlands; OXCARRE, Department of Economics, University of Oxford, Oxford, OX1 3UQ UK

**Keywords:** Exhaustible resources, Hotelling rule, Efficiency, Carbon tax, Climate rent, Differential game, Open-loop Nash equilibrium, Subgame-perfect Nash equilibrium, HARA production functions, C73, H30, Q32, Q37, Q54

## Abstract

Industria imports oil, produces final goods and wishes to mitigate global warming. Oilrabia exports oil and buys final goods from the other country. Industria uses the carbon tax to impose an import tariff on oil and steal some of Oilrabia’s scarcity rent. Conversely, Oilrabia has monopoly power and sets the oil price to steal some of Industria’s climate rent. We analyze the relative speeds of oil extraction and carbon accumulation under these strategic interactions for various production function specifications and compare these with the efficient and competitive outcomes. We prove that for the class of HARA production functions, the oil price is initially higher and subsequently lower in the open-loop Nash equilibrium than in the efficient outcome. The oil extraction rate is thus initially too low and in later stages too high. The HARA class includes linear, loglinear and semi-loglinear demand functions as special cases. For non-HARA production functions, Oilrabia may in the open-loop Nash equilibrium initially price oil lower than the efficient level, thus resulting in more oil extraction and climate damages. We also contrast the open-loop Nash and efficient outcomes numerically with the feedback Nash outcomes. We find that the optimal carbon tax path in the feedback Nash equilibrium is flatter than in the open-loop Nash equilibrium. It turns out that for certain demand functions using the carbon tax as an import tariff may hurt consumers’ welfare as the resulting user cost of oil is so high that the fall in welfare wipes out the gain from higher tariff revenues.

## Introduction

An oil-exporting cartel such as the OPEC can exert monopoly power on the world market, especially if the price elasticity of oil demand is not too high. This monopoly power can result in significantly different oil extraction patterns relative to a perfectly competitive market. A key factor that determines the effect of the exporter’s monopoly power is the nature of the importer’s oil demand, since the price elasticity of oil demand is for most demand functions not constant. However, many studies when modeling oil extractions either choose iso-elastic demand or choose a particular specification of the demand function (typically, linear demand) for reasons of convenience/simplicity to make the models tractable. A key result in the literature on oil extraction is that with zero extraction costs and iso-elastic demand, the monopolist oil extraction is efficient and coincides with what would prevail in a competitive market [18]. In that case, the oil price increases according to the Hotelling rule at a rate equal to the market rate of interest [10]. For non-iso-elastic demand, the oil price path can be steeper or flatter, depending on the demand’s functional form. It is thus important to analyze the robustness of results to the chosen specific functional forms for the production function and oil demand. This allows us to break down the way various demand specifications affect the bias of the exporter’s monopoly power on the oil extraction rate and thus on carbon emissions.

The monopolist’s oil extraction rate also plays a prominent role in the context of climate policies. As there is no global agreement on battling climate change, increasingly the developed countries are beginning to implement their own carbon emission reduction policies, while the oil-exporting nations are opposed to such measures. There is a range of studies modeling strategic interaction between a monopolist oil exporter who sets the oil price and an importer who combats climate change by setting a carbon tax [17, 19, 20, 24] and surveyed in [15]. In equilibrium, the oil-importing country uses the carbon tax as an import tariff to capture some of the monopolist’s rents, while the oil-exporting country marks up his price so that he can capture a part of the carbon tax revenue collected by the oil-exporting country.

Liski and Tahvonen [14] explicitly analyze such strategic interactions. Their main results are twofold: First, the subgame-perfect oil extraction path is flatter than in the efficient outcome; second, this path is also flatter than in the pure cartel outcome where no carbon tax is levied. Furthermore, they find that the level of damages due to global warming has a significant effect on equilibrium dynamics. The level of damages determines whether the import tariff or the Pigouvian environmental tax is the dominant component of the importer’s carbon tax, and hence, whether the carbon tax decreases or increases over time. The authors even find that for very high damages, the tariff component may be negative—a subsidy. However, Liski and Tahvonen [14] restrict their analysis to linear demand functions and parameterizations that lead to interior solutions and thus keep some oil left in situ. As we will see, these assumptions do end up affecting their result.

Our contribution is to investigate the generality of these results to a variety of specifications of production functions and oil demand. Our framework of analysis is partial equilibrium, since we abstract from saving, investment and capital accumulation and take the interest rate as given.^1^ Although we offer formal propositions where it is feasible, we also have to resort to numerical simulations to gain insights. For this purpose, we use an illustrative calibration of our model to real-world values. We obtain the following findings.

First, we recall that unlike the case of zero extraction cost, a constant price elasticity of oil demand and no climate concerns [5, 18], if oil extraction costs are nonzero and increase as remaining oil reserves diminish, monopolistic extraction is never efficient. We establish the nature of the inefficiency for the open-loop Nash equilibrium too.

Second, we prove that for the class of HARA production functions and associated oil demand functions, the oil price is initially higher and subsequently lower in the open-loop Nash equilibrium than in the efficient outcome. The oil extraction rate and carbon emissions are thus initially too low and in later stages too high relative to the efficient levels. The HARA class includes linear [14], loglinear and semi-loglinear demand functions as special cases. This proof also holds for the case of a cartel.

Third, we show that on the other hand, a shifted loglinear demand function can lead Oilrabia to initially price oil lower in open-loop Nash equilibrium than in the efficient level, thus resulting in more oil extraction and climate damages. This establishes that there exist non-HARA demand functions that yield opposite results without violating any arbitrage conditions. Lewis et al. [13] have demonstrated a similar result in a context without carbon taxation: If the price elasticity of demand increases in oil consumption, monopolistic extraction is initially faster than the efficient rate. Our study thus differs in that we allow for carbon pollution and global warming and the consequent need for the oil-importing country to impose a carbon tax.^2^

Fourth, our simulation results indicate that the feedback Nash equilibrium leads initially to flatter oil price paths than the efficient outcome. We establish numerically that there is also a downward initial bias in oil extraction rates and carbon emissions for the feedback Nash equilibrium. The consumer oil price increases slower in the feedback Nash equilibrium than in the open-loop Nash equilibrium, and our simulations also indicate that the optimal carbon tax path will always be higher in the feedback Nash equilibrium than in the open-loop Nash equilibrium. This occurs because in the feedback Nash equilibrium the oil-importing region increases the carbon tax in order to capture the scarcity rents of the oil-exporting country.

Fifth, we find that for oil demand specifications which asymptotically lead to full oil exhaustion, the level of carbon damages does not influence the carbon tax dynamics.^3^ For such oil demand functions, the monopolist exporter earns significant amounts of scarcity rent, and thus, the carbon tax is used primarily as a tariff so that the importer can capture some of these rents back. However, as Oilrabia and Industria compete to capture each other’s rents, they raise the combined user cost of oil so high that the resulting welfare loss to the consumers is larger than the extra-gain from stolen scarcity rents.

Finally, our simulations indicate that for a reasonable calibration of the model, no matter how high the intensity of global warming damages, the oil-importing country will always impose a positive import tariff on top of the carbon tax in the feedback Nash equilibrium. Thus, the result of Liski and Tahvonen [14] that in some cases, the importer may have an incentive to subsidize oil consumption in order to ease the burden caused by the high prices set by the oil-exporting countries does not seem to be relevant.

We must make two important provisos to our findings. The first one is that we abstract from political economy issues. For example, oil exporters such as Saudi Arabia may have a desire to charge a higher export price of oil if their populations are sizeable and need to be pacified with transfers. Saudi Arabia would then be hurt if the oil-importing countries levy carbon taxes, especially if a lot of these are shifted to Saudi Arabia. Also, oil-importing countries may hesitate to fully internalize the social cost of carbon if they fear the income distributions of regressive carbon taxes, but we abstract from such political economy effects too. The second proviso is that we focus at open-loop and feedback Nash equilibrium outcomes and abstract from open-loop and feedback Stackelberg equilibrium outcomes for a supply side consisting of a cartel of monopolistic oil exporters and a competitive fringe of smaller oil exporters.^4^

Although our analysis has antecedents in the economics of natural resources (e.g., [5, 13, 14, 17–20, 24]), it is important to realize that our analysis also has roots in the industrial organization and international trade literature. For example, Brander and Spencer [3] examine how a tariff on imports produced by a foreign Cournot–Nash oligopoly is affected by linear and other plausible demand specifications and Brander and Spencer [4] also analyze how export subsidies might affect the optimal import tariff and how in a context of three countries export subsidies might affect the optimal import tariff or the nature of the Nash equilibrium in tariffs and subsidies between the three countries. Our analysis builds on this literature by explicitly taking account of the scarcity of fossil fuel and allow for both the Pigouvian and the import tariff motive for carbon taxation. Our work is also related to Mrázová and Neary [16] and Xie [21] who identify which demand function features, especially whether demand is super- or sub-modular indicated by the sign of the so-called super-elasticity, affect results in industrial organization and international trade. These insights are more relevant in Stackelberg than in Nash setups, so are of less relevance for our present analysis of the monopolist’s price markup and the importer’s carbon tax.

The outline of the paper is as follows. Section 2 sets up the model of the oil-importing and oil-exporting blocks of countries and discusses the differential game that is played between them. Section 3 derives the open-loop Nash equilibrium outcome, compares this with the efficient and competitive outcomes and proves that for the class of HARA production functions, the resulting oil price path is flatter and the oil extraction rate path is steeper in open-loop Nash equilibrium than in the efficient outcome. Section 4 derives the subgame-perfect Nash equilibrium outcome (also known as the feedback Nash equilibrium). Section 5 offers some illustrative simulations that confirm our proposition on HARA production functions, provides an example of demand for which the monopolist initially extracts less than the efficient rate, and more generally highlights the differences between the open-loop and subgame-perfect equilibrium and the efficient and competitive outcomes. Section 6 concludes with a summary of results and suggestions for further research.

## The Model

Our model describes two countries or blocks of countries. One country called “Industria” does not have oil, but imports $R$ units of oil from the other country called “Oilrabia” and uses it to produce final goods according to the concave production function $F(R)$ with $F(0)=0$, $F^{\prime }(R)>0$ and $F^{\prime \prime }(R)<0.$ All other factors of production (labor, land, etc.) are fixed and subsumed in the production function. $C$ units of final goods are bought by Industria and $C^{*}$ units by Oilrabia for consumption purposes. A further $G(S)R$ units of final goods are bought by Oilrabia for extraction purposes, where $S$ is the stock of oil reserves. Marginal oil extraction costs rise as less oil reserves are left and less accessible fields have to be explored, so $G^{\prime }(S)<0.$ Furthermore, we suppose that marginal oil extraction costs are convex, $G^{\prime \prime }(S)>0.$ Instantaneous world goods market equilibrium requires that

1 $$\begin{aligned} F(R)=C+C^{*}+G(S)R. \end{aligned}$$

Oilrabia lives from its oil wealth, has monopoly power in the world market for oil and has utilitarian preferences.^5^ The government of Oilrabia has a given initial stock of oil $S_0$, which it manages optimally to maximize the present discounted value of its consumption stream which corresponds to the present value of its oil profits. Industria also has utilitarian preferences and is concerned with the present value of its consumption stream. It also levies a specific carbon tax $\tau$ to limit the damages from global warming. We are concerned with strategic interactions between Industria and Oilrabia, where the former sets the carbon tax and the latter sets the oil price.

### Demand for Oil and Private Consumption in Industria

Firms in Industria operate under perfect competition and maximize profits $\varPi \equiv F(R)-(p+\tau )R,$ taking the before-tax price of oil $p$ and the specific carbon tax $\tau$ as given. Here profits include wage income and land rental income. We measure oil in tons of carbon, which implies a carbon emission coefficient of unity. The efficiency condition for an interior solution reads $F^{\prime }(R)=p+\tau$, where $F^{\prime }(R)$ is the marginal product of oil. It gives oil demand as a decreasing function of the user cost of oil, namely $R=R(p+\tau )$. Consumers in Industria obtain income from final goods production and get carbon tax revenues rebated in a lump-sum fashion, so that consumption is given by

2 $$\begin{aligned} C= & {} \varPi +\tau R=F( {R(p+\tau )} )-pR(p+\tau )\equiv C(p,\tau ), \nonumber \\ C_p= & {} -\left[ {1+\frac{\tau }{p+\tau }\varepsilon } \right] R<0,\quad C_\tau =-\frac{\tau }{p+\tau }\varepsilon R<0, \end{aligned}$$

where $\varepsilon \equiv -(p+\tau )R^{\prime }(p+\tau )/R(p+\tau )>0$ is the price elasticity of oil demand. A higher producer price of oil cuts consumption directly as oil inputs are more costly and indirectly via the downward effect on oil demand if and only if the carbon tax is positive. We also give the full partial derivatives of consumption with respect to the tax and price so that we can gain more intuition about the relationship between consumption, price, tax and the elasticity of demand.

### The Government of Industria

The stock of carbon in the atmosphere, $E$, increases with carbon emissions, so that

3 $$\begin{aligned} \dot{E}=R(p+\tau ),\quad E(0)=E_0 \hbox { given}, \end{aligned}$$

where we abstract from natural decay of the stock of atmospheric carbon. Global warming damages are given by the function $D(E)$ with $D^{\prime }(0)>0,\;D^{\prime \prime }(E)\ge 0$ and $D(E_0 +S_0 )<\infty$. The objective of the government of Industria at time $t$ is to choose the carbon tax to maximize the present discounted value of private consumption of different generations of households minus global warming damages:

4 $$\begin{aligned} V(t)=\int _t^\infty {e^{-\rho (s-t)}\left[ {C( {p(s),\tau (s)} )-D( {E(s)} )} \right] \mathrm{d}s} , \end{aligned}$$

subject to the dynamics of the atmospheric stock of carbon (3), where $\rho >0$ is the constant rate of time preference. Linear preferences imply zero intergenerational inequality aversion, so that social welfare across generations is utilitarian. The social rate of discount is used by the government of Industria to discount welfare of future generations.^6^ Although all oil extraction takes place outside of Industria’s borders, Industria’s government is able to observe the remaining oil stock $S$. The dynamics of oil extraction follow from:

5 $$\begin{aligned} \dot{S}=-R(p+\tau )\ge 0,\quad S(t)\ge 0,\quad S(0)=S_0 \quad \hbox {given}. \end{aligned}$$

For all instants of time, the stock of carbon in the atmosphere is the initial stock plus cumulative oil depletion, $E(t)=E_0 +S_0 -S(t),$ so that, formally, only the stock of atmospheric carbon or the stock of in situ oil needs to be considered as a state variable.

### The Government of Oilrabia

The government of Oilrabia takes account of oil demand from Industria and chooses oil prices to maximize the present discounted value from the stream of current and future oil profits,

6 $$\begin{aligned} V^{*}(t)=\int _t^\infty {e^{-\rho ^{*}(s-t)}\left[ {p(s)R( {p(s)+\tau (s)} )-G( {S(s)} )R( {p(s)+\tau (s)} )} \right] \mathrm{d}s} , \end{aligned}$$

subject to the dynamics of oil depletion (5). Here $\rho ^{*}>0$ is Oilrabia’s rate of time preference. We will suppose that Industria and Oilrabia employ the same rate of time preference: $\rho =\rho ^{*}$. From (1) and (2), we get Oilrabia’s consumption rate, $C^{*}=F(R)-C-G(S)R=pR-G(S)R$. Oilrabia’s government maximizes (6) or equivalently the present value of Oilrabia’s stream of current and future consumption levels, $V^{*}(t)=\int _t^\infty {e^{-\rho (s-t)}C^{*}(s)\mathrm{d}s}$. Our general equilibrium model is similar to the two-country model of an oil exporter and an oil importer analyzed by Liski and Tahvonen [14] if the production function $F(R)$ is replaced by the utility function $U(R)$ with $U^{\prime }>0\;\hbox {and }U^{\prime \prime }<0.$ In their model demand for oil comes from households setting $U^{\prime }(R)=p+\tau$ and in our model, it comes from firms producing final goods setting $F^{\prime }(R)=p+\tau$. Of course, their model can have $U(0)<0$, whereas we have $F(0)=0$.

## Efficient and Open-Loop Nash Equilibria

We first consider the efficient equilibrium where a social planner maximizes the welfare sum of Oilrabia and Industria. We then compare it to the open-loop Nash equilibrium where each of the countries takes the time path of the other country’s policy as given when setting its own policy. This requires commitment to announced policies and is distinct from the feedback Nash equilibrium where the countries cannot pre-commit to a time path, but instead base their strategies on the remaining oil stock $S$ and pollution stock $E$. We will derive the feedback Nash equilibrium in Sect. 4.

### Efficient Equilibrium

In the efficient equilibrium, the social planner sets the user cost of oil, which is defined as $q\equiv p+\tau$, such that it maximizes total global consumption less the environmental damages, subject to the resource extraction constraint in (2). The Hamiltonian for the social planner is then $H\equiv F(R(q))-G(S)R(q)-D(E)-\mu R(q)$, where the co-state variable $\mu$ corresponds to the sum of the social cost of carbon and the Hotelling rent. We assume $F^{\prime }(0)>G(S_0 )+D^{\prime }(E_0 )/\rho$ in order to avoid the possibility of zero extraction throughout. Indeed, if the inequality would not hold, even the marginal revenues of low initial extraction would not cover the lowest possible sum of marginal oil extraction cost and the social cost of carbon.

#### **Proposition 1**

It is optimal to have nonzero oil extraction rates forever in the efficient outcome.

#### *Proof*

It is optimal to have an interior solution $R(t)>0$ for all $t\ge 0$, because if $R(t)=0$ for all $t\ge T$ for some $T\ge 0$, then this $T$ is the optimal stopping time and the Hamiltonian should equal zero at $T$ so that $F(0)=D^{\prime }(E_0 +S_0 -S(T))=0$. This is ruled out by the assumption $D^{\prime }(E)>0$ for all $E>0$. Moreover, there cannot be positive extraction followed by zero extraction and then positive extraction again, which follows from strictly positive discounting and the fact that in the zero extraction phase, the state of the system is not changing. $\square$

Given Proposition 1, the optimality conditions are:

7 $$\begin{aligned} \frac{\mathrm{d}H}{\mathrm{d}q}\!=\!\left[ {F^{\prime }\left( {R^{\prime }(q)} \right) -G(S)-\mu } \right] \frac{\mathrm{d}R}{\mathrm{d}q}=0\quad \hbox {and}\quad \rho \mu -\dot{\mu }=\frac{\mathrm{d}H}{\mathrm{d}S}={D}'(E)-{G}'(S)R.\quad \end{aligned}$$

Simplifying the optimality conditions (7), we can rewrite them to obtain an ordinary differential equation for the user cost of oil $q$:

7′ $$\begin{aligned} \dot{q}=\rho \left[ {q-G(S)-{D}'(E)/\rho } \right] . \end{aligned}$$

The consumer price of oil, also called the user cost of oil, can be decomposed into two components: the producer price of oil (which increases according to the Hotelling rule) and the Pigouvian carbon tax (which is equal to the sum of discounted marginal damages from emitting one ton of carbon). This result is fairly straightforward and has been shown in many previous studies, but we reproduce it here for the ease of comparison with the open-loop and feedback Nash equilibrium results that we discuss later on.

#### **Proposition 2**

The optimal oil extraction rate is monotonically decreasing over time and vanishes only asymptotically in the efficient outcome. The corresponding stock of oil reserves gradually falls to zero if $F^{\prime }(0)-G(0)>\frac{D^{\prime }(E_0 +S_0 )}{\rho }$ (full exhaustion). Otherwise, it diminishes to a strictly positive steady-state level (partial exhaustion).

#### *Proof*

From the optimality conditions (7), we can calculate how much oil is left in situ in the long run. We first rewrite (7) in terms of the marginal productivity of oil to obtain $$\dot{F}^{\prime }(R)=\rho \left[ {F^{\prime }(R)-G(S)-D^{\prime }(E_0 +S_0 -S)/\rho } \right] .$$ Note that $$G(S(t))+D^{\prime }\left( {E_0 +S_0 -S(t)} \right) $$ is increasing over time, because the oil stock is decreasing over time. This implies that the optimal extraction rate is monotonically decreasing toward zero; otherwise, there would exist $$t_1 <t_2 $$ with $$R(t_1 )=R(t_2 )$$ and $$\dot{R}(t_1 )<0,\;\dot{R}(t_2 )>0.$$ However, that is impossible, as according to (7′) that would imply that $$G(S(t))+D^{\prime }\left( {E_0 +S_0 -S(t)} \right) $$ would be decreasing over time. Thus, to examine the long-run steady state of the in-situ oil stock, we take the limit of Eq. (7′) as $$R$$ and $$\dot{R}$$ approach 0 and derive the following expression:

7′′ $$\begin{aligned} F^{\prime }(0)-G(S)=D^{\prime }(E_0 +S_0 -S)/\rho . \end{aligned}$$

Since the extraction rate monotonically decreases toward zero, expression (7′′) thus gives us the stock of oil at which further oil extraction is no longer profitable. The left-hand side gives the marginal benefit net of the cost of extracting a marginal unit of fossil fuels, while the right-hand side gives the total discounted marginal global warming damages from doing so. We can then identify two separate cases. The first case prevails if $F^{\prime }(0)-G(0)>\frac{D^{\prime }(E_0 +S_0 )}{\rho }$ and occurs, for example, if $F^{\prime }(0)=\infty$ and $G(0)<\infty .$ In this first case, the productivity of the marginal unit of fossil fuels is so high that extraction will always be profitable. This will lead to oil extraction that will go on forever, the stock of oil reserves approaching zero in the limit. The second case prevails if $F^{\prime }(0)-G(S)=\frac{D^{\prime }(E_0 +S_0 -S)}{\rho }$ and has a solution in the range $0\le S<S_0$. Then, in the long run, $S$ will be left in the ground. $\square$

The two cases described in Proposition 2 are relevant not only just for the efficient equilibrium but also for the open-loop and the feedback Nash equilibrium outcomes. In fact, as Liski and Tahvonen [14] demonstrate, in the long run, both the feedback and the open-loop Nash equilibrium outcomes converge to the efficient stock of oil reserves. Thus, the amount of oil left in situ remains the same across the different equilibrium outcomes. Which of the above two cases occurs depends on the nature of oil demand and the parameterization.

### Open-Loop Nash Equilibrium: Carbon Taxation in Industria

We now move on to calculating the open-loop Nash equilibrium, first looking at Industria’s problem. Industria maximizes its welfare (4) subject to (2). Its current value Hamiltonian therefore reads $H\equiv F(R(p+\tau ))-G(S)R-D(E)-\mu R(p+\tau )$, where $\mu \ge 0$ is Industria’s social cost of carbon. The optimality conditions for the carbon tax, the adjoint equation for the social cost of carbon and the transversality condition are, respectively,

8 $$\begin{aligned} \frac{\partial H}{\partial \tau }= & {} \left[ {F^{\prime }\left( {R(p+\tau )} \right) p-\mu } \right] R^{\prime }(p+\tau )=0,\quad \rho \mu -\dot{\mu }=\frac{\partial H}{\partial E}=D^{\prime }(E),\nonumber \\&\mathop {\lim }\limits _{t\rightarrow \infty } \;e^{-\rho t}\mu (t)=0. \end{aligned}$$

We assume that the world market price of oil is such that we have an interior solution. This assumption will be verified in due course. The first part of (8) tells us that the optimal carbon tax must be set to Industria’s social cost of carbon, $\tau =\mu$. The third part of (8) is the transversality condition, which is necessary due to the characteristics of our optimization problem (e.g., Michel 1982). It follows that the second part of (8) can be integrated, using the transversality condition, to give the optimal carbon tax as the discounted sum of all future marginal damages from global warming:

9 $$\begin{aligned} \tau (t)=\int _t^\infty {e^{-\rho (s-t)}D^{\prime }( {E(s)} )\mathrm{d}s} ,\quad t\ge 0. \end{aligned}$$

### Open-Loop Nash Equilibrium: Oil Pricing by Oilrabia

Oilrabia’s optimization problem is to maximize (6) subject to (5). Its current value Hamiltonian reads $H^{*}\equiv \left[ {p-G(S)-\lambda ^{*}} \right] R(p+\tau )$, where $\lambda ^{*}\ge 0$ is the marginal value of oil reserves to Oilrabia (also called the scarcity rent). The optimality conditions for an interior solution and the transversality condition read:

10 $$\begin{aligned} \frac{\partial H^{*}}{\partial p}= & {} R(p+\tau )+\left[ {p-G(S)-\lambda ^{*}} \right] R^{\prime }(p+\tau )=0, \nonumber \\&\rho \lambda ^{*}-\dot{\lambda }^{*}=\frac{\partial H^{*}}{\partial S}=-G^{\prime }(S)R(p+\tau ),\quad \mathop {\lim }\limits _{t\rightarrow \infty } \;e^{-\rho t}\lambda ^{*}(t)=0. \end{aligned}$$

The first two equations of (10) imply that the optimal price of oil must equal the sum of the oil extraction cost, the scarcity rent of oil and the carbon tax, all multiplied by a monopoly markup:

11 $$\begin{aligned} p+\tau =\frac{G(S)+\lambda ^{*}+\tau }{1-1/\varepsilon }. \end{aligned}$$

In a competitive market $(\varepsilon \rightarrow \infty )$, the oil price is simply set to the sum of the extraction cost and the scarcity rent and thus (11) becomes $p=G(S)+\lambda ^{*}$. An alternative way of writing (11) gives (11′) with the market price of oil as the monopoly markup on the extraction cost (the first term on the right-hand side) and the scarcity rent (the first term on the right-hand side) plus the capture of the climate rent (the second term):

11′ $$\begin{aligned} p=\frac{G(S)+\lambda ^{*}}{1-1/\varepsilon }+\frac{\tau }{\varepsilon -1}. \end{aligned}$$

Capture of climate rent is more substantial if Oilrabia has more monopoly power on the oil market, which is the case if the price elasticity of oil demand $\varepsilon$ is relatively low. Integrating the second part of (10) and using the transversality condition give the scarcity rent as the present value of all future reduction extraction costs resulting from keeping an extra unit of oil in the earth:

12 $$\begin{aligned} \lambda ^{*}(t)=-\int _t^\infty {e^{-\rho (s-t)}G^{\prime }( {S(s)} )R(s)\mathrm{d}s} . \end{aligned}$$

Having derived the dynamics for Oilrabia’s price and Industria’s tax, we now combine them to find equilibrium conditions.

### Open-Loop Nash Equilibrium Outcome

It is easiest to analyze the open-loop Nash equilibrium outcome in terms of oil reserves $S$ and the user cost of oil $q$. Combining (10) and (8), the open-loop Nash outcome is described by the dynamic system specified by Eq. (5) and

13 $$\begin{aligned} \dot{q}= & {} \frac{\rho \left[ {\left( {1-\frac{1}{\varepsilon (q)}} \right) q-G(S)} \right] -{D}'(E_0 +S_0 -S)}{2-\frac{{R}''(q)R(q)}{{R}'(q)^{2}}}\nonumber \\= & {} \frac{\rho \left[ {\left( {1-\frac{1}{\varepsilon (q)}} \right) q-G(S)} \right] -{D}'(E_0 +S_0 -S)}{1-\frac{1}{\varepsilon (q)}+\frac{\varTheta (q)}{\varepsilon (q)}}, \end{aligned}$$

where $\varTheta (q)\equiv \frac{q\varepsilon ^{\prime }(q)}{\varepsilon (q)}$ defines the elasticity of demand or the so-called super-elasticity (e.g., [16]). For iso-elastic demand, we have $\varTheta =0$ and the denominator boils down to the familiar monopoly markup (see Sect. 3.5.1). For the class of HARA production functions, we have $\varTheta >0$ which increases the denominator and tends to flatten the price path relative to the case of iso-elastic oil demand (see Sect. 3.5.2). There exist non-HARA production functions for which $\varTheta <0$ and the denominator tends to increase so that the price path steepens relative to the case of iso-elastic oil demand (see Sect. 3.5.3).

The efficient outcome is described by Eq. (5) and

14 $$\begin{aligned} \dot{q}=\rho \left[ {q-G(S)} \right] -D^{\prime }(E_0 +S_0 -S), \end{aligned}$$

and the competitive market outcome by Eq. (5) and

15 $$\begin{aligned} \dot{q}=\rho \left[ {q-G(S)} \right] . \end{aligned}$$

The cartel or monopolist outcome (where Industria does not set a carbon tax) follows as a special case of the open-loop Nash equilibrium outcome:

13′ $$\begin{aligned} \dot{q}=\frac{\rho \left[ {\left( {1-\frac{1}{\varepsilon (q)}} \right) q-G(S)} \right] }{2-\frac{{R}''(q)R(q)}{{R}'(q)^{2}}}=\frac{\rho \left[ {\left( {1-\frac{1}{\varepsilon (q)}} \right) q-G(S)} \right] }{1-\frac{1-\varTheta (q)}{\varepsilon (q)}}. \end{aligned}$$

Note that in any equilibrium, we must have

16 $$\begin{aligned} G(S)<p,\quad D^{\prime }(E)/\rho \le \tau . \end{aligned}$$

The first inequality is needed to have nonnegative profits from oil extraction. The second inequality follows from the fact that the carbon tax is monotonically non-decreasing, because if it were decreasing, it would become negative eventually, which contradicts that there is a cost rather than a benefit associated with carbon accumulation. Hence, in each of the efficient, monopolistic and competitive outcomes, the user cost of oil is increasing at a rate no greater than the social rate of discount. Another reason for considering only such price paths is to exclude arbitrage, as pointed at by Dasgupta and Heal [5] for the case without extraction cost and global warming damages.

### Comparing the Open-Loop Nash and Efficient Equilibria

We now compare the results under the various outcomes described by (13), (13′), (14) and (15). To provide some initial insights, we first consider in Sect. 3.5.1, the case of iso-elastic demand as this produces the well-known result that monopolistic extraction is efficient [18] and thus provides a useful benchmark. If oil demand is not iso-elastic, we see from (13) that the solution becomes less trivial and depends on the sign of the super-elasticity and thus on the degree the consumers can substitute away from oil as the price rises, and thus how much scarcity rent the monopolist can capture. We spend the remainder determining the way in which various production function specifications affect equilibrium dynamics. In Sect. 3.5.2, we generalize the benchmark case of iso-elastic demand to the much more general class of HARA production functions, which captures most of the commonly used demand functions, and prove that for this class initial oil prices in the open-loop equilibrium outcome are too low and later on are too high relative to the efficient outcome. Section 3.5.3 discusses an example of a non-HARA production function, which is useful as this can give the opposite to our main result on HARA production functions.

#### Case: Iso-elastic Oil Demand

We have for this case $F(R)=\frac{R^{1-\sigma }}{1-\sigma },\;R=p^{-1/\sigma },\;2-\frac{R^{\prime \prime }R}{R^{\prime 2}}=1+\sigma$, constant elasticity $\varepsilon =\frac{1}{\sigma }$, and $\varTheta =0$, with $1>\sigma >0$. Equation (13) for the user oil cost dynamics under open-loop Nash equilibrium becomes $\dot{q}=\frac{\rho \left[ {(1-1/\varepsilon )q-G(S)-{D}'(E_0 +S_0 -S)/\rho } \right] }{1-1/\varepsilon }$. In (8), we have shown that the open-loop Nash equilibrium tax is the Pigouvian tax which evolves according to $\dot{\tau }=\rho \tau -{D}'(E_0 +S_0 -S)$. Hence, we get the dynamics of the producer price of oil:

17 $$\begin{aligned} \dot{p}=\rho \left( {p-\frac{G(S)}{1-1/\varepsilon }} \right) -\frac{{D}'(E_0 +S_0 -S)}{\varepsilon -1} \end{aligned}$$

The last term in the right-hand side of (17) indicates that the oil-exporting region uses its monopoly market power to capture a part of the climate rent by charging higher prices. Let us now consider some special outcomes. First, if extraction costs and climate damages are zero, (17) boils down to $\dot{p}=\rho p$, so that the open-loop Nash equilibrium and cartel outcomes are Pareto efficient and correspond to the competitive outcome (cf. [18]); Eqs. (13), (13′), (14) and (15) all boil down to $\dot{p}=\rho p$. Second, the open-loop Nash equilibrium is inefficient if there are extraction costs even if there are no climate damages, as Eq. (17) boils down to $\dot{p}=\rho \left( {p-\frac{G(S)}{1-1/\varepsilon }} \right)$ which only represents the efficient outcome if the oil market is competitive ($\varepsilon \rightarrow \infty$). Third, if the world oil market is competitive, $\varepsilon \rightarrow \infty$, Eq. (17) boils down to the Hotelling dynamics $\dot{p}=\rho \left[ {p-G(S)} \right]$ for the producer price of oil and thus we have $\dot{q}=\rho \left[ {q-G(S)-{D}'(E_0 +S_0 -S)/\rho } \right]$ for the dynamics of the consumer price of oil. Hence, the return on leaving an extra-unit of oil in the ground (the capital gains) equals the interest on the net revenue from extracting and selling an extra-unit of oil. The open-loop outcome then corresponds to the efficient outcome (14) which coincides with the competitive outcome (15) only if damages from global warming are zero.

#### HARA Class Demand

Let us consider the class of HARA demand functions. This class of production functions is given by

18 $$\begin{aligned} F(R)=\frac{1-\varphi }{\varphi }\left[ {\left( {\frac{\psi R}{1-\varphi }+\chi } \right) ^{\varphi }-\chi ^{\varphi }} \right] ,\quad \hbox {where } \psi >0,\;\chi \ge 0\;\;\hbox { and }\;\; \varphi >0. \end{aligned}$$

##### **Proposition 3**

For the class of HARA production functions with elastic demand $(\varepsilon >1)$, initial oil extraction along the open-loop Nash equilibrium is less than that in the efficient equilibrium.

##### *Proof*

The demand function is $R(q)=(y-\chi )\left( {\frac{1-\varphi }{\psi }} \right)$, where $y\equiv \left( {\frac{q}{\psi }} \right) ^{\frac{1}{\varphi -1}}>0$. Strictly positive demand requires $(y-\chi )(1-\varphi )>0$. The price elasticity of demand is $\varepsilon (q)=\frac{y}{(1-\varphi )(y-\chi )}$. Since we restrict the analysis to elastic demand $(\varepsilon >1)$, we need the condition $\varphi y+(1-\varphi )\chi >0$ to be satisfied. As $\chi \ge 0$, the inequality $\varphi y+(2-\varphi )\chi >0$ must hold too. We also have $\frac{R^{\prime \prime }(q)R(q)}{R^{\prime }(q)^{2}}=\frac{(2-\varphi )(y-\chi )}{y}$. Define $V^{N}\equiv G(S^{N})+D^{\prime }(E^{N})/\rho$ and $V^{E}\equiv G(S^{E})+D^{\prime }(E^{E})/\rho$, where the superscripts $N$ and $E$ refer to the open-loop Nash and the efficient equilibrium, respectively. Substituting the above expressions for $\varepsilon (q),\frac{R^{\prime \prime }(q)R(q)}{R^{\prime }(q)^{2}}$ in terms of $y$ and $G(S^{N})+D^{\prime }(E^{N})/\rho =V^{N}$ into Eq. (13) for the open-loop Nash equilibrium, then yields:

19 $$\begin{aligned} \dot{q}^{N}=\rho \left[ {\frac{(1-\varphi )\chi +\varphi y}{(2-\varphi )\chi +\varphi y}q^{N}-\frac{y}{(2-\varphi )\chi +\varphi y}V^{N}} \right] . \end{aligned}$$

Using (7′) or (14) and substituting $G(S^{E})+D^{\prime }(E^{E})/\rho =V^{E}$, we get for the efficient equilibrium:

19′ $$\begin{aligned} \dot{q}^{E}=\rho (q^{E}-V^{E}). \end{aligned}$$

With the conditions and results discussed so far, we proceed with a proof by contradiction. Suppose therefore, contrary to the claim of this proposition, that initial extraction along the open-loop Nash equilibrium is larger than in the efficient equilibrium. Since, as has been proven by Liski and Tahvonen [14], in the long run, the same amount of oil is cumulatively extracted for both equilibria, the initial price along the open-loop Nash equilibrium is smaller than in the efficient equilibrium and there must be an instant of time, $T$, where $q^{N}(T)=q^{E}(T)=q(T)$ with $\dot{q}^{N}(T)>\dot{q}^{E}(T)$. In other words, the open-loop Nash price path must intersect the efficient price path from below and must thus be steeper at the point of intersection. So, we have from Eqs. (19) and (19′) that

20 $$\begin{aligned} \frac{\dot{q}^{N}(T)}{\rho }= & {} \frac{(1-\varphi )\chi +\varphi y(T)}{(2-\varphi )\chi +\varphi y(T)}q(T)-\frac{y(T)}{(2-\varphi )\chi +\varphi y(T)}V^{N}(T)>\frac{\dot{q}^{E}(T)}{\rho }\nonumber \\= & {} q(T)-V^{E}(T)). \end{aligned}$$

Hence, using the condition $\varphi y+(2-\varphi )\chi >0$ discussed above, we see from (20) that the inequality $\chi q(T)<\left[ {(2-\varphi )\chi +\varphi y(T)} \right] V^{E}(T)-y(T)V^{N}(T)$ must hold. Note that up to time $T$, more oil is extracted in the open-loop Nash than in the efficient equilibrium and that both the extraction cost and climate damages are monotonically decreasing in the stock of oil. Therefore, the total social cost of oil at time $T$ must be higher for the open-loop case, i.e., $V^{N}(T)>V^{E}(T)$. Using these last two inequalities, we get $\chi q(T)<\left[ {(2-\varphi )\chi +(\varphi -1)y(T)} \right] V^{E}(T)$. Since the set of inequalities in (16) implies that the inequality $V^{E}<q$ must hold, we have $\chi q(T)<\left[ {(2-\varphi )\chi +(\varphi -1)y(T)} \right] q(T)$. Given that $q(T)>0$ and $\chi \ge 0,$ it must hold that $(1-\varphi )\left[ {y(T)-\chi } \right] <0$. This violates condition 1. $\square$

This proposition establishes that for all members of the HARA class of production functions, the extraction rates in the open-loop Nash equilibrium are initially too low and later on are too high compared with the efficient outcome. Of course, oil prices are then initially too high and later on too low in the open-loop Nash equilibrium. This result also holds for the monopolist. Indeed, if this were not the case, it would be unprofitable for the monopolist to enter the market. In that sense, monopolistic oil barons are the conservationist’s best friend.

The class of HARA production functions has super-elasticity $\varTheta (q)=\frac{\chi }{1-\varphi }\left[ (q/\psi )^{\frac{1}{\varphi -1}}-\chi \right] ^{-1}.$ If $\chi >0,$ then $\varTheta (q)>0$ as positive marginal demand requires $\left[ {(q/\psi )^{\frac{1}{\varphi -1}}-\chi } \right] (1-\varphi )>0.$ If $\chi =0,$ then $\varTheta (q)=0.$ Thus, the members of the HARA class of production functions have a positive (or zero for the case of iso-elastic demand) super-elasticity, which tends to flatten the price path (13) relative to the case of iso-elastic oil demand. Proposition 3 proves that the extraction rate in open-loop Nash equilibrium is in fact slower than the efficient rate.

It is important to realize that the class of HARA production functions is quite general, since it includes many familiar production functions as special cases. Table 1 presents five special cases of the HARA production functions: quadratic, exponential, Cobb–Douglas, power and logarithmic production functions. These correspond to, respectively, linear, semi-loglinear, iso-elastic, shifted loglinear and shifted unit-elastic oil demand specifications. For each of the HARA production functions, the associated demand function, the elasticity and the parameter restrictions that must hold are given in Table 1 too.

Section 5 uses the linear, semi-loglinear and iso-elastic demand specifications in our numerical simulations of the open-loop Nash equilibrium, feedback Nash equilibrium and efficient outcomes. The simulations for shifted loglinear and shifted unit-elastic demand specifications are not presented as they give qualitatively similar insights.

**Table 1:** Various HARA production functions

Production function	Demand function	Demand elasticity	Parameter restrictions
$F(R)=-\frac{1}{2}\left( {\chi -\psi R} \right) ^{2}+\frac{1}{2}\chi ^{2}$	$R=(\chi \psi -q)/\psi ^{2}$	$\varepsilon =\frac{q}{\chi \psi -q}$	$\varphi =2, \chi >0, q<\chi \psi$
(quadratic)	(linear)		
$F(R)=1-e^{-\psi R}$	$R=-\frac{1}{\psi }\ln \left( {\frac{q}{\psi }} \right)$	$\varepsilon =-\frac{1}{\ln \left( {\frac{q}{\psi }} \right) }$	$\varphi \rightarrow -\infty , \chi =1$
(exponential)	(semi-loglinear)		
$F(R)=\frac{1-\varphi }{\varphi }\left( {\frac{\psi R}{1-\varphi }} \right) ^{\varphi }$	$R=\frac{1-\varphi }{\psi }\left( {\frac{q}{\psi }} \right) ^{\frac{1}{\varphi -1}}$	$\varepsilon =\frac{1}{1-\varphi }$	$\chi =0, \varphi >0$
(Cobb–Douglas)	(loglinear/iso-elastic)		
$F(R)=\frac{1-\varphi }{\varphi }\left[ {(R+\chi )^{\varphi }-\chi ^{\varphi }} \right]$	$R=(\frac{q}{1-\varphi })^{\frac{1}{\varphi -1}}-\chi$	$\varepsilon =\frac{(\frac{q}{1-\varphi })^{\frac{1}{\varphi -1}}}{(1-\varphi )\left[ {(\frac{q}{1-\varphi })^{\frac{1}{\varphi -1}}-\chi } \right] }$	$0<\varphi <1, \chi >0, \psi =1-\varphi$
(power)	(loglinear with shift in oil demand)		
$F(R)=\log (R+\chi )-\log (\chi )$	$R=\frac{1}{q}-\chi$	$\varepsilon =\frac{1}{1-\chi q}$	$\varphi \rightarrow 0, \psi =1, \chi q<1$
(logarithmic)	(shifted unit-elastic)		

#### Non-HARA Class Demand

We now examine a class of non-HARA production functions with a negative super-elasticity to show that it is possible that the open-loop Nash equilibrium is bad for the environment. Consider $F(R)=\chi R+\frac{R^{1-1/\phi }}{1-1/\phi }$ with $\phi >1$, and $\chi >0$, which is an alternative power production function to the one reported in Table 1 as the shift occurs in the oil price rather than in oil demand. This gives rise to the shifted loglinear oil demand curve, $R=(q-\chi )^{-\phi }$ with $q>\chi$. It has elasticity $\varepsilon =\left( {\frac{q}{q-\chi }} \right) \phi >0$. This demand function has a negative super-elasticity, $\varTheta (q)=-\frac{\chi q}{\phi (q-\chi )}<0$. Hence, from (13), this tends to steepen the price path relative to the case of iso-elastic oil demand. In fact, Eq. (13) becomes

21 $$\begin{aligned} \dot{q}=\frac{\rho \left[ {(\phi -1)q+\chi -\phi G(S)} \right] -\phi D^{\prime }(E_0 +S_0 -S)}{\phi -1}. \end{aligned}$$

We can rule out $$\dot{q}>\rho q$$ on the basis of arbitrage arguments [5]. Combining $$\dot{q}\le \rho q$$ and the open-loop Nash equilibrium first-order conditions, we obtain the following necessary condition: $$\frac{\rho \chi }{q\phi -1}-\frac{\rho }{q(\phi -1)}(\chi -G(S)-D^{\prime }(E)/\rho )<0$$. As damages are increasing in $$E$$ and extraction costs are decreasing in $$S,$$ the lowest value of extraction cost is at $$S_0 $$ and damages at $$E_0 $$. Therefore, the necessary condition can be transformed to:

21′ $$\begin{aligned} \frac{1}{\phi -1}\left[ {\chi -\phi \left[ {G(S_0 )+D^{\prime }(E_0 )/\rho } \right] } \right] <0. \end{aligned}$$

Thus, $\chi$ has to be small enough to not violate the arbitrage condition (21) but large enough so that the production function differs significantly from the classic iso-elastic case (for which the monopolist extracts slower than the efficient rate). It is not possible to derive precise analytical conditions for when the open-loop Nash equilibrium is initially less conservative than the efficient outcome. Note, however, that using conditions (13) and (5), one can numerically solve for time paths of $S$ and $q$ and then use (9) to find the equilibrium carbon tax and oil price. In Sect. 5, we perform such a numerical analysis for a variety of demand specifications and offer an example which demonstrates that the monopolist can be less conservationist than the efficient outcome.

In fact, our simulations establish that the open-loop Nash equilibrium outcome can lead to a faster initially extraction rate than the efficient rate. We cannot make any definitive conclusions, because we can also find other examples of shifted loglinear demand function where in the open-loop equilibrium, the monopolist is initially more conservative than in the efficient case.

## Feedback Nash Equilibrium

We assume that both Industria and Oilrabia have perfect information on both remaining oil reserves and the atmospheric carbon stock at each instant of time.^7^ Hence, the optimal carbon tax imposed by Industria and the world market price of oil determined by Oilrabia can both be represented as a function of merely remaining oil reserves, since the stock of atmospheric carbon is the sum of the initial carbon stock and all oil burnt so far. We denote the equilibrium price and the equilibrium tax by $p(S)$ and $\tau (S)$ and their derivatives by $p_S (S)$ and $\tau _S (S)$.

### The Feedback Rule for the Optimal Carbon Tax

The Hamilton–Jacobi–Bellman (HJB) equation for Industria is

22 $$\begin{aligned} \rho V(S)=\mathop {\max }\limits _\tau \left[ {F(R(p+\tau ))-p(S)R(p+\tau )-D(S_0 +E_0 -S)-V_S (S)R(p+\tau )} \right] ,\nonumber \\ \end{aligned}$$

where $V(S)$ is Industria’s value function. In a feedback Nash equilibrium, Industria takes the price of oil as a given function of oil reserves and atmospheric carbon $p(S)$. The optimality condition $[F^{\prime }(R(p+\tau ))-p]R^{\prime }(\hbox {p}+\tau )=V_S (S))R^{\prime }(p+\tau )$ gives Industria’s feedback rule for the optimal carbon tax:

23 $$\begin{aligned} \tau =V_S (S)=\tau (S). \end{aligned}$$

The carbon tax $\tau$ is thus set to the shadow price of the oil stock. Substituting (23) into (22) and differentiating both sides with respect to the oil stock, we obtain the equation describing the dynamics of the carbon tax:

24 $$\begin{aligned} \tau _S R(p+\tau )=-\rho \tau -p_S (S)R(p+\tau )+D^{\prime }(S_0 +E_0 -S). \end{aligned}$$

The carbon tax can then be decomposed as a sum of the environmental tax $\tau ^{E}$ and the import tariff $\tau ^{T}$. The environmental tax is the classic Pigouvian tax which is set to the total discounted marginal damages from pollution, while the import tariff is the strategic component which Industria uses to steal Oilrabia’s monopoly rent. The dynamics of these components follow from:

24′ $$\begin{aligned} \tau _S^E R(p+\tau )=-\rho \tau ^{E}+D^{\prime }(S_0 +E_0 -S)\quad \! \hbox { and }\quad \! \tau _S^T R(p\!+\!\tau )=-\rho \tau ^{T}-p_S (S)R(p\!+\!\tau ). \end{aligned}$$

### The Optimal Feedback Rule for the Oil Price

Oilrabia maximizes the present discounted value of profits from oil extraction (6) subject to oil demand, carbon accumulation (3) and oil depletion (5) and given the policy rule for Industria’s carbon tax $\tau (S)$. The HJB equation for Oilrabia is

25 $$\begin{aligned} \rho V^{*}(S)=\mathop {\max }\limits _p \left\{ {\left[ {p-G(S)} \right] R(p+\tau )-V_S^*(S)R(p+\tau )} \right\} , \end{aligned}$$

where $V^{*}(S)$ is the value function for Oilrabia. The first-order condition gives the optimal oil price:

26 $$\begin{aligned} p=\frac{G(S)-V_S^*(S)}{1-1/\varepsilon (p+\tau )}+\frac{\tau }{\varepsilon (p+\tau )-1}. \end{aligned}$$

The first term in the right-hand side of expression (26) is the monopoly markup on the sum of extraction cost and the scarcity rent. The markup increases if oil demand is less elastic. The second term in the right-hand side of (26) depends on the size of the carbon tax levied by Industria and reflects the extent to which Oilrabia can capture part of Industria’s climate rent, which is easier if Oilrabia has more monopoly power on the world oil market.

To derive the dynamics of Industria’s oil price, we substitute (26) into (25) and differentiate both sides with respect to $S$, leading to the following equation

27 $$\begin{aligned} (p_S +\tau _S )R(p+\tau )\left( {\frac{R(p+\tau ){R}''(p+\tau )}{{R}'(p+\tau )^{2}}-2} \right) =\rho \left[ {p-\frac{p+\tau }{\varepsilon (p+\tau )}-G(S)} \right] . \end{aligned}$$

### Equilibrium

The feedback Nash equilibrium solves for the optimal rule for Industria (24) and Oilrabia (27). Combining them, we get the equilibrium condition for the user cost of oil

28 $$\begin{aligned} q_S R(q)\left( {\frac{R(q){R}''(q)}{{R}'(q)^{2}}-3} \right) =\rho \left[ {q\left( {1-\frac{1}{\varepsilon (q)}} \right) -D^{\prime }(E_0 +S_0 -S)/\rho -G(S)} \right] .\quad \end{aligned}$$

As can be seen, similar to the open-loop Nash equilibrium, the demand function specification plays a key role in the nature of the feedback Nash equilibrium. An analytic solution is feasible if preferences are quadratic and extraction cost is linear by guessing that value functions are quadratic and solving with the method of undetermined coefficients. For more sophisticated demand specifications, one normally needs to find a fixed point in the policy reaction functions and value functions. However, for a problem with a single state variable, there are easier solution methods.

### Time-Domain Representation and Comparison with the Open-Loop Nash Equilibrium Outcome

We begin by demonstrating that we can express $p(S)$ and $\tau (S)$ as optimal paths $p(t)$ and $\tau (t)$, without any information loss. Formally, the feedback Nash equilibrium implies that the players do not pre-commit to optimal policy paths and instead the policies are expressed as functions of the state variable. However, an interior solution is characterized by positive oil demand, i.e., $R(t)>0$ for all $t\ge 0.$ This implies that the oil stock $S$ is a monotonically decreasing function of time $\dot{S}=-R<0$. Therefore, for every instant of time $t$, there is a corresponding unique value of $S(t)\in (S_0 ,0)$. Thus, the solutions to the feedback equilibrium $p(S(t))$ and $\tau (S(t))$ can also be expressed simply as optimal equilibrium paths: $p(t)$ and $\tau (t)$.

We now present the time-domain representation of the feedback equilibrium conditions (24) and (27). In addition to simplifying the numerical solution of the feedback equilibrium, expressing the optimal conditions in the time domain allows for easier comparison with the open-loop Nash equilibrium. Utilizing $-\tau _S R=\dot{\tau }$ and $-p_S R=\dot{p}$, the feedback equilibrium carbon tax dynamics in (24) becomes:

29 $$\begin{aligned} \dot{\tau }=\rho \tau -{D}'(E_0 +S_0 -S)-\dot{p}. \end{aligned}$$

Compared to the open-loop Nash equilibrium carbon tax in (9), an extra-term is added to the feedback equilibrium expression—the change in the oil price $-\dot{p}<0$. Hence, the feedback carbon tax path is flatter than the open-loop one. The tax can then be decomposed into the environmental tax $\tau ^{E}$ and an import tariff $\tau ^{T}$:

25′ $$\begin{aligned} \dot{\tau }^{E}=\rho \tau ^{E}-{D}'(E_0 +S_0 -S),\quad \dot{\tau }^{T}=\rho \tau ^{T}-\dot{p}. \end{aligned}$$

Note that the open-loop Nash equilibrium carbon tax is the same as the environmental component of the feedback Nash carbon tax. In the open-loop Nash equilibrium, Industria has to pre-commit to a tax and does not consider the response of Oilrabia—thus simply setting the carbon tax to the sum of the discounted marginal damages. In the feedback equilibrium, Industria adds a tariff component to steal some of the scarcity rent from Oilrabia.

We can also rewrite the feedback equilibrium condition of Oilrabia as

30 $$\begin{aligned} \dot{p}+\dot{\tau }=\frac{\rho \left[ {(p+\tau )\left\{ {1-1/\varepsilon (p+\tau )} \right\} -\tau -G(S)} \right] }{2-R(p+\tau )R^{\prime \prime }(p+\tau )/\left( {R^{\prime }(p+\tau )} \right) ^{2}}. \end{aligned}$$

This expression is similar to the open-loop Nash equilibrium dynamics in (13). Since in equilibrium $\rho \tau >D^{\prime }(E)$, we conclude that the path for the user cost of oil indeed tends to be flatter in the feedback Nash equilibrium than in the open-loop Nash equilibrium outcome. To make solving the equilibrium easier, we combine (29) and (30), representing the equilibrium through the dynamics of the combined user cost of oil:

31 $$\begin{aligned} \dot{q}= & {} \frac{\rho \left[ {\left\{ {1-1/\varepsilon (q)} \right\} q-G(S)-D^{\prime }(E_0 +S_0 -S)/\rho } \right] }{3-R(q)R^{\prime \prime }(q)/\left( {R^{\prime }(q)} \right) ^{2}} \nonumber \\= & {} \frac{\rho \left[ {\left\{ {1-1/\varepsilon (q)} \right\} q-G(S)-D^{\prime }(E_0 +S_0 -S)/\rho } \right] }{2-\frac{1-\varTheta (q)}{\varepsilon }}. \end{aligned}$$

Comparing (31) with (13), we see that the denominator has increased by unity and thus the dynamics of the consumer price have a smaller derivative which suggests that the corresponding price path will be flatter. Of course, this does not constitute a proof that the paths of the user cost of oil will be flatter as this would require insight into the solution of the model. Our simulations, however, do confirm that the consumer price path is indeed flatter in feedback Nash equilibrium than in open-loop Nash equilibrium.

We collect some of our results on the feedback Nash equilibrium in the following proposition.

#### **Proposition 4**

For the feedback Nash equilibrium, the optimal carbon tax consists of the sum of a Pigouvian component and an import tariff on oil from Oilrabia. The monopoly markup on the sum of extraction cost and the scarcity rent increases if oil demand is less elastic. Oilrabia captures part of Industria’s climate rent, and more so if it has more monopoly power on the world oil market.

The feedback Nash equilibrium solution can now be obtained by numerically solving (5) and (31) for the time paths of $$S$$ and $$q$$. From these, one can calculate the time paths for the carbon stock $$E=E_0 +S_0 -S,$$ the carbon tax $$\tau =[ {\dot{q}+D^{\prime }(E)} ]/\rho $$ (from 31), and the market price of oil $$p=q-\tau $$. We can calculate the pure Pigouvian component of the carbon tax as $$\tau ^{P}(t)\equiv \int _t^\infty {e^{-\rho (s-t)}D^{\prime }\left( {E(s)} \right) \mathrm{d}s} $$, upon which the import tariff component can be found as $$\tau (t)-\tau ^{P}(t)$$. The export markup for oil follows from $$p-G(S)-\lambda ^{**}$$, where the scarcity rent is calculated as $$\lambda ^{**}(t)=-\int _t^\infty {e^{-\rho (s-t)}G^{\prime }\left( {S(s)} \right) R(s)\mathrm{d}s}$$. These price and tax components can then be used to demonstrate how the demand specification influences the amount of monopolist rent that can be stolen by the importer and vice versa.

Solving this feedback Nash equilibrium problem is relatively straightforward, due to the fact that our model collapses to only one state variable (the oil stock). If instead two or more state variables were required to describe the state of the world, we would have to explicitly solve for optimal strategies as functions of the states. For example, if we had included atmospheric decay of carbon in our model, the atmospheric carbon stock would no longer be the complement of the oil stock, and the two countries would base their strategies on both stocks. Similarly, if we would have allowed for capital accumulation in the model, the optimal strategies would also be a function of the capital stock.

## Illustrative Numerical Simulations

We supplement our analytic results by illustrating the efficient, the open-loop and the feedback Nash equilibrium outcomes with some numerical solutions. For these purposes, we use the functional forms and calibration reported in Table 2. All our emission variables are expressed in 1000 Giga tons of carbon (GtC), and prices and costs are expressed in $1000 USD per ton carbon (tC).

We adopt a linear extraction cost function with an initial stock of oil reserves of 10 GtC. Extraction costs rise from an initial value of $300 to $1150 per tC when reserves are exhausted corresponding to $30 and $115 per barrel of oil, and following the [11] long-term cost curve. The initial carbon stock is set to its pre-industrial level of 800 GtC. Global warming damages are given by a quadratic function, with $$\kappa >0$$ as the damage intensity parameter. We consider quadratic, exponential and Cobb–Douglas production functions, which are all members of the HARA class (see Table 1), as well as a non-HARA production function. They lead to, respectively, linear, semi-loglinear, iso-elastic and shifted loglinear oil demand functions. Further calibration details are reported in “Appendix 1.”

In the remainder of this section, we present the numerical solutions for various demand specifications; “Appendix 2” gives details of our numerical procedure. We begin in Sect. 5.1 by replicating the results of Liski and Tahvonen [14] by solving the model for quadratic production functions with linear oil demand. Section 5.2 shows that the qualitative insights do not change for exponential production functions with semi-loglinear oil demand.

**Table 2:** Calibration

	Functional form	Parameter values
Extraction cost	$G(S)=\gamma _1 +\gamma _2 (S_0 -S)$	$\gamma _1 =0.3,\gamma _2 =0.085,S_0 =10$
Damages	$D(E)=\kappa E^{2}$	$E_0 =8,\kappa =0.00004$
Quadratic production	$F(R)=\chi \psi R-\psi ^{2}R^{2}/2$	$\psi =0.855,\beta =2.61$
(linear demand)		
Exponential production	$F(R)=\frac{A}{\psi }(1-e^{-\psi R})$	$\psi =3.672,A=.7430$
(semi-loglinear oil demand)		
Cobb–Douglas production	$F(R)=\frac{1-\varphi }{\varphi }\left( {\frac{\psi R}{1-\varphi }} \right) ^{\varphi }$	$\varphi =0.35,\psi =13.94$
(iso-elastic demand)		
Non-HARA production	$F(R)=\chi R+\frac{R^{1-1/\phi }}{1-1/\phi }$.	$\phi =2.7, \chi =1$
(shifted loglinear demand)		

**Fig. 1:**
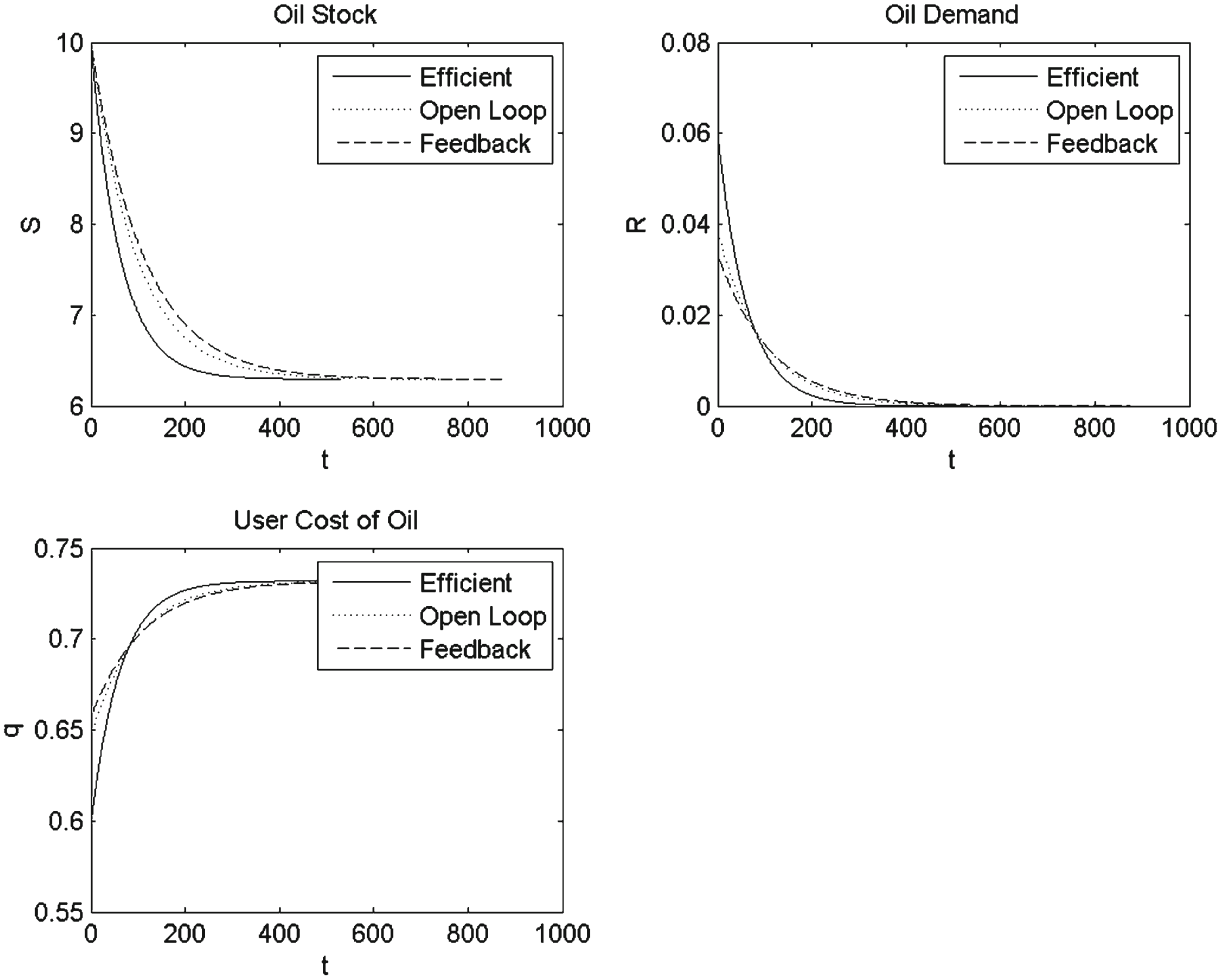
Simulations with quadratic production function and linear oil demand

We solve the model for various levels of the damage intensity parameter, $\kappa$, demonstrating that given a reasonable calibration, the damage parameter has a relatively minor effect on the resource extraction rate. Section 5.3 presents the results for the benchmark case of a Cobb–Douglas production function with loglinear or iso-elastic oil demand. Here the government of Industria in the desire to capture the exporter’s scarcity rent raises the tariff, to which Oilrabia’s government responds by raising the price even higher, causing ever more damage to the consumer. Section 5.4 discusses the example of the non-HARA production function with shifted loglinear oil demand discussed in Sect. 3.5.3 in which the monopolist initially extracts more oil than the efficient rate.

### Quadratic Production Function and Linear Oil Demand

We first consider the case of a quadratic production function and linear oil demand, which is the functional form used by Liski and Tahvonen [14]. Figure 1 compares time paths for oil reserves and the rate of oil extraction for the various outcomes. It confirms that in the open-loop Nash equilibrium, oil is initially extracted more slowly than in the efficient outcome, but faster than in the feedback Nash equilibrium. However, in the long run, the stock of oil reserves converges to the same level for these three outcomes. The user cost of oil in the open-loop Nash equilibrium outcome is initially higher and later on lower than the efficient outcome reflecting the slower depletion of oil reserves, thus confirming proposition 3 and the results of Liski and Tahvonen [14]. This bias in the extraction rate and slowing down of the depletion of the stock of oil reserves is bigger in the feedback Nash equilibrium than in the open-loop Nash equilibrium, thus confirming the insight of proposition 4 that the price path in the feedback Nash equilibrium is flatter than in the open-loop Nash equilibrium.

Table 3 gives the welfare for the various outcomes and indicates, not surprisingly, that Industria does worst in the feedback Nash equilibrium outcome relative to both open-loop and efficient welfare levels. The open-loop Nash equilibrium obviously yields higher welfare for Industria than the efficient outcome. Interestingly, Oilrabia does better in the open-loop Nash equilibrium, where it can change its markup and earn more profit on oil than the efficient outcome where it cannot, but does worse in the feedback Nash equilibrium where Industria can use the carbon tax as a tariff to steal some of Oilrabia’s monopolist rents.

**Table 3:** Welfare in the various outcomes

Oil demand	Industria’s welfare			Oilrabia’s profit		
	Efficient	Open-loop	Feedback	Efficient	Open-loop	Feedback
Linear	$-$0.188	$-$0.242	$-$0.195	0.276	0.309	0.250
Semi-loglinear	$-$0.183	$-$0.237	$-$0.189	0.279	0.313	0.252
Iso-elastic	10.887	8.797	8.075	2.802	3.552	2.938

**Fig. 2:**
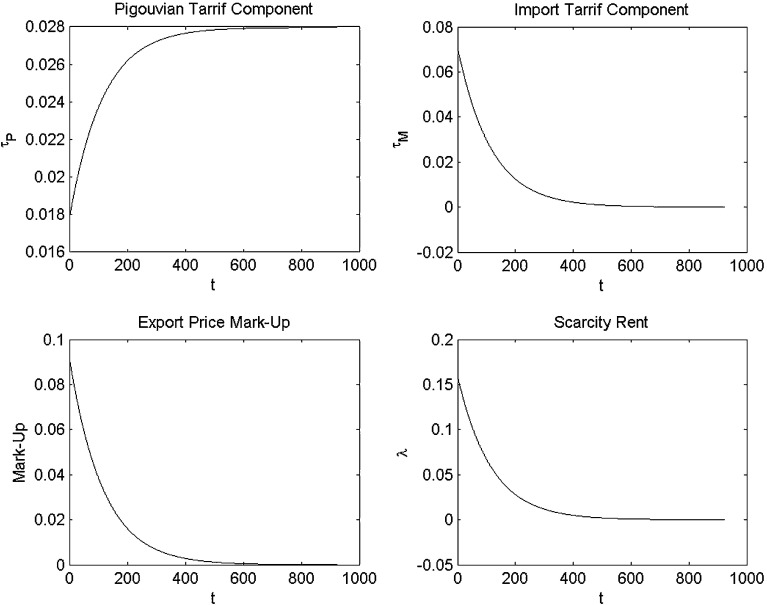
Decomposing the carbon tax and the oil price in feedback Nash equilibrium with linear oil demand

Figure 2 decomposes the feedback Nash equilibrium paths for the optimal carbon tax set by Industria into its Pigouvian tax and its import tariff component and similarly decomposes the optimal oil price set by Oilrabia into the scarcity rent and the export price markup. The first panel of Fig. 2 shows that the Pigouvian tax component rises over time as the stock of atmospheric carbon rises and marginal climate damages go up. The second panel of Fig. 2 also indicates that the import tariff component falls over time as the potential oil rents that can be captured decline as oil reserves fall. Effectively, Industria uses its monopsony market power to extract rents from Oilrabia with a high initial import tariff component that decreases over time. Since the calibration we use assumes fairly mild damages, the import tariff component of the carbon tax dominates the Pigouvian tax components.

The fourth and third panels of Fig. 2 indicate that the scarcity rent $\lambda$ falls over time and that the export price markup falls over time too, since the extraction cost rises and the cartel loses its monopoly power.

**Fig. 3:**
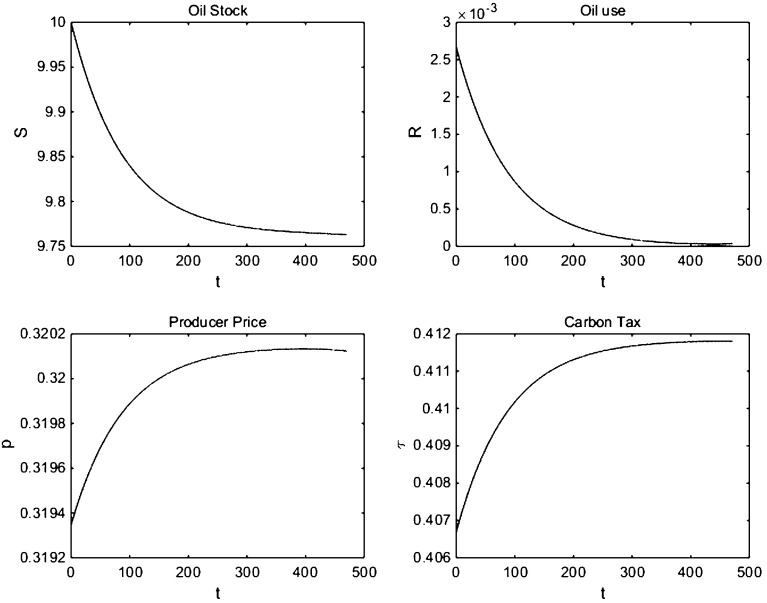
Simulation with linear oil demand and high climate damages

**Fig. 4:**
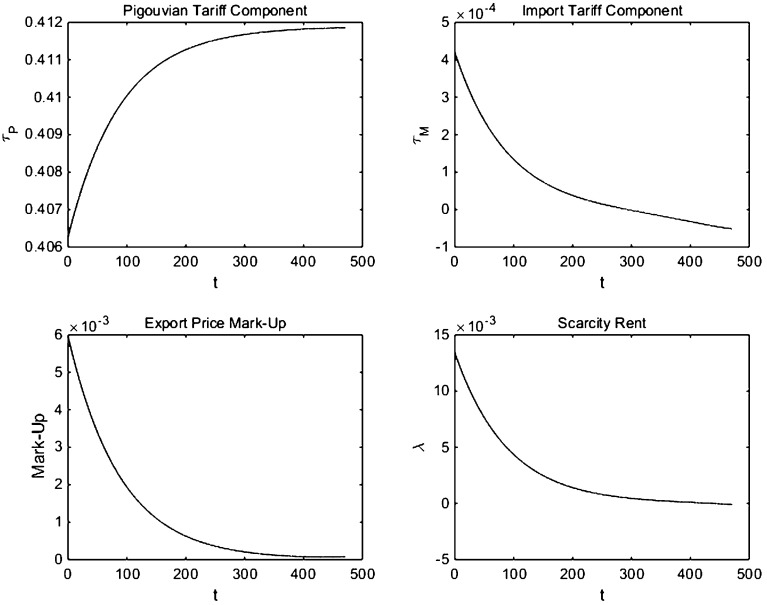
Decomposition of the carbon tax and the oil prices for linear demand with high climate damages

To see how the severity of climate damages affects the components of the equilibrium, Figs. 3 and 4 report the results for high-severity climate damages. Similar to Liski and Tahvonen [14], we find that with medium climate damages, the Pigouvian carbon tax component is fairly large, as indicated in the first panel of Fig. 4. Industria still has a little market power to extract some rents from Oilrabia, and the import tariff component is dominated by the Pigouvian component. This result can be observed in Fig. 4 where the import tariff (second panel) and markup (third panel) are much smaller than the environmental component of the carbon tax. Note that the larger Pigouvian tax component increases over time, while the import tariff decreases as scarcity rents converge to zero. Thus, as can be seen in the bottom right panel of Fig. 3, the total optimal carbon tax increases over time with high climate damages. This contrasts with the decreasing carbon tax in Fig. 1 when climate damage intensity is lower and the carbon tax is primarily used as an import tariff.

However, we could not find a damage intensity that is high enough to ensure that the optimal feedback Nash carbon tax becomes smaller than the Pigouvian tax. Instead, for a very high climate damage intensity, oil extraction becomes too harmful, and Industria sets a prohibitive carbon tax such that no oil is extracted. This result differs from [14]. Recall that in that study, for very high values of climate damage intensity, Industria sets the carbon tax below the optimal Pigouvian level, thus imposing an import subsidy (i.e., a negative tariff). This contradiction is mainly driven by our calibration. Thus, for our calibration, we can conclude that no matter how high the climate damages, the importer will always add an import tariff on top of the Pigouvian carbon tax.

### Exponential Production Function with Semi-loglinear Oil Demand

We now explore the case of an exponential production function with semi-loglinear oil demand. Semi-loglinear and linear demand specifications lead to fairly similar equilibrium extraction paths. The key difference is quantitative. The initial equilibrium oil extraction rate and oil price are the same for both demand specifications, But the amounts left in the ground differ as can be seen from Table 4.

**Table 4:** Time at which 95 % of oil is extracted and untapped oil reserves at that time

Oil demand	95 % Extraction time			Untapped oil reserves
	Efficient	Open-loop	Feedback	
Linear	192.3	300.4	345.8	6.295
Semi-loglinear	204.3	321.2	370.2	6.179
Iso-elastic	116.3	258.6	489.7	0
Non-HARA: Shifted unit-elastic	114.5	116.8	144.6	0

As we mentioned earlier, the long-run stock of untapped oil reserves is only approached asymptotically. For all equilibrium outcomes, some extraction will take place. As can be seen from Table 4, with semi-loglinear oil demand, Oilrabia leaves more oil in situ than with linear demand.

Table 3 indicates that with semi-loglinear demand, Industria’s welfare is still worse in the feedback Nash equilibrium than in the open-loop Nash equilibrium and a fortiori worse than in the efficient outcome. Oilrabia’s welfare is again worse than the efficient outcome in the feedback Nash equilibrium, but better than the efficient outcome in the open-loop Nash equilibrium.

### Cobb–Douglas Production Function and Iso-elastic Oil Demand

**Fig. 5:**
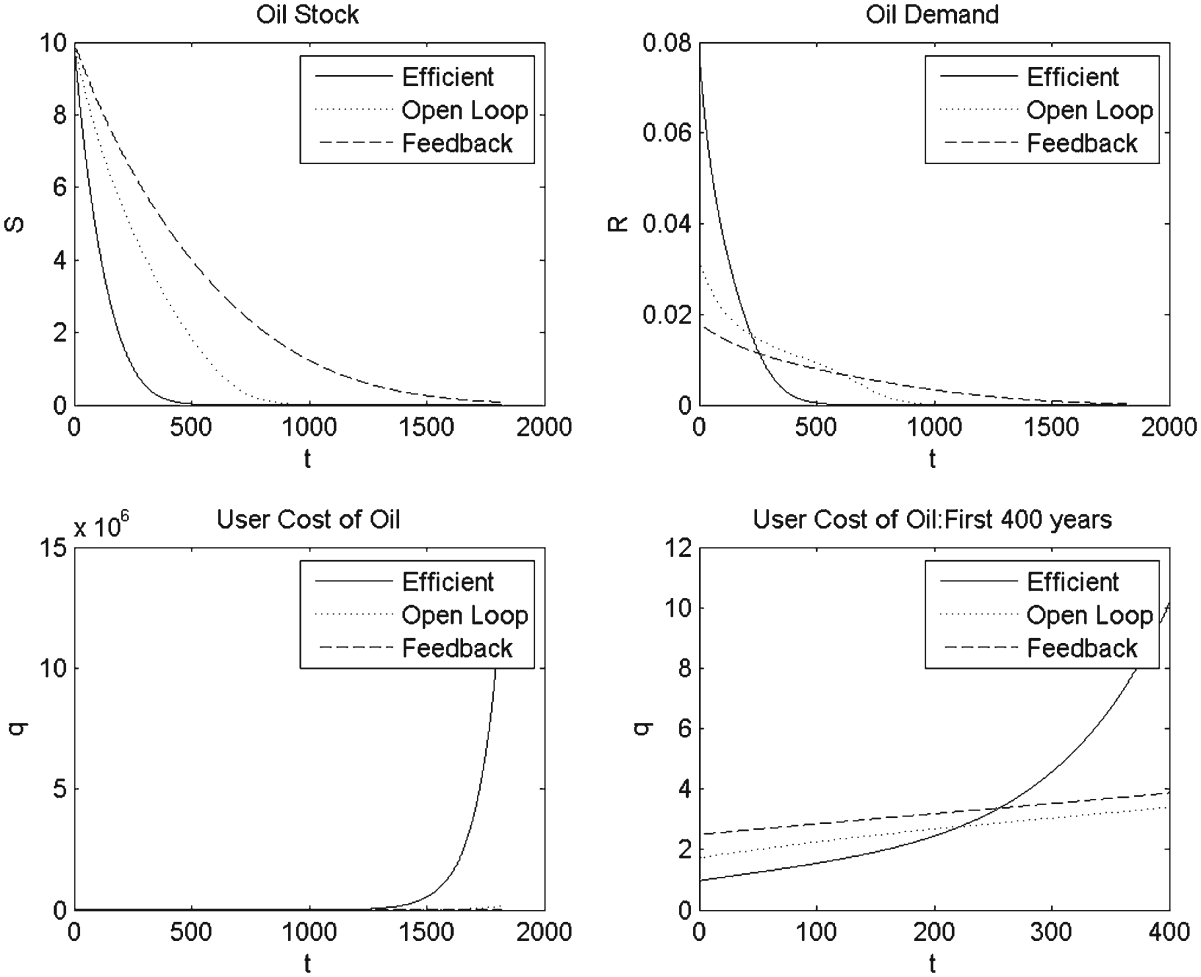
Simulations with iso-elastic oil demand

**Fig. 6:**
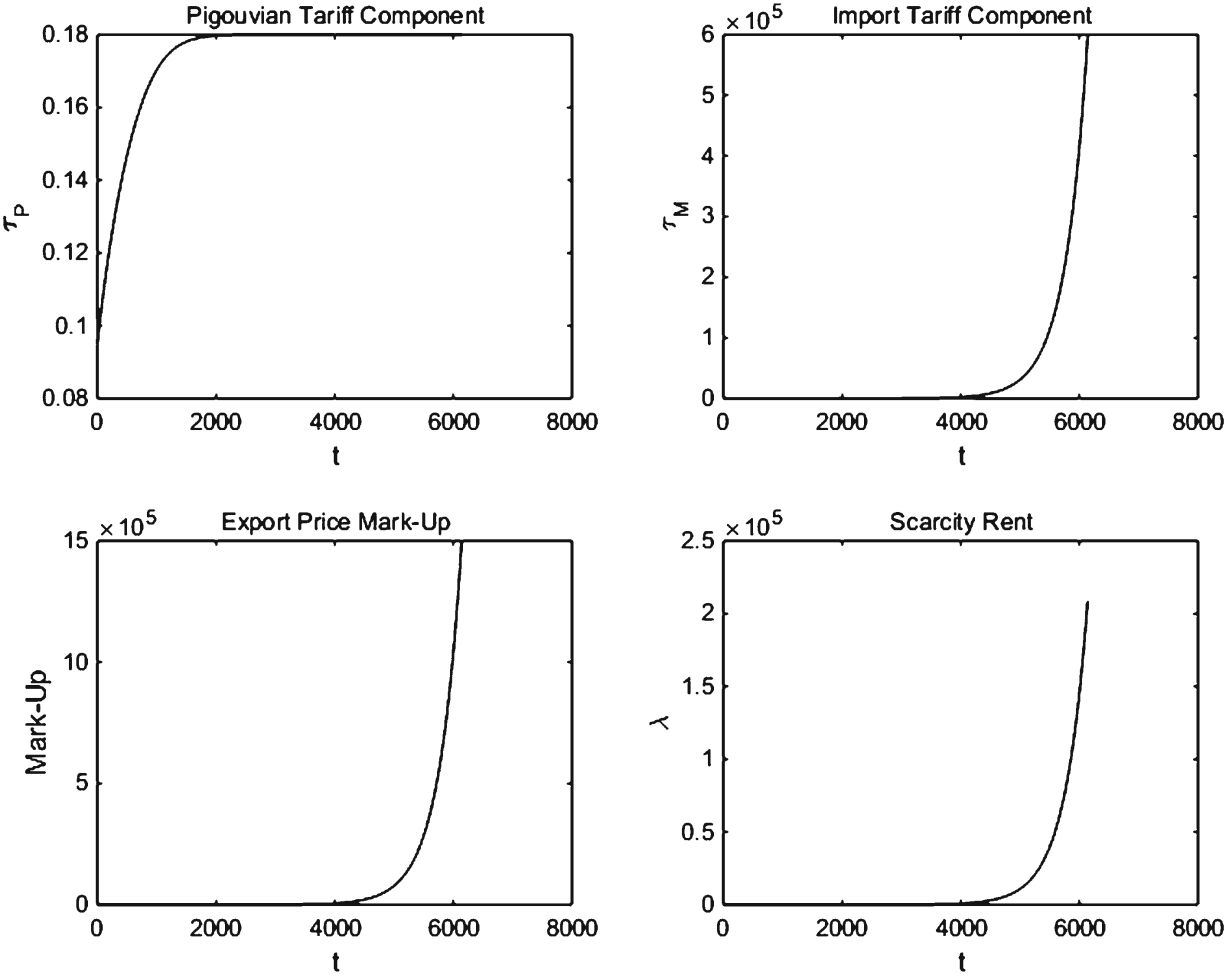
Decomposition of carbon tax and oil price for the feedback Nash equilibrium with iso-elastic oil demand

We now examine the benchmark case of a Cobb–Douglas production function giving loglinear or iso-elastic oil demand. Note that solving this case is less straightforward than with linear and semi-loglinear oil demand, since now all oil is depleted asymptotically (full exhaustion) as with loglinear oil demand the productivity of the marginal unit of oil exceeds the extraction cost of the marginal unit of oil. In contrast, with linear and semi-loglinear oil demand, some oil is left in situ (partial exhaustion). It is for this reason that studying loglinear or iso-elastic oil demand is often avoided sticking to functional forms which lead to partial exhaustion and hence an interior solution (e.g., [17, 24]). We resolve this issue with a slightly more complicated numerical procedure (“Solving for Equilibria with Infinite Marginal Production at Zero” section of Appendix 2) and give simulations for iso-elastic oil demand in Figs. 5 and 6.

The main result of Liski and Tahvonen [14] holds in the sense that the path of the user cost of oil is less steep for both the feedback and the open-loop Nash equilibrium than for the efficient case and that in the feedback Nash equilibrium, the stock of oil reserves is depleted more slowly than in the open-loop Nash equilibrium and a fortiori than in the efficient outcome. The results differ in the redistribution of rents. As mentioned earlier, with iso-elastic oil demand, marginal productivity of oil is infinite at zero use, and hence in the long run, the price goes to infinity instead of converging to a finite level. As the stock of oil reserves vanishes, Oilrabia increases the price of oil, and there are always incentives for Industria to increase the tariff on oil. We have solved for the feedback equilibrium for a range of damage intensity levels, and this result holds consistently. Hence, no matter how severe the climate damage, the import tariff component always increases over time and always dominates the Pigouvian tax component. Thus, with iso-elastic oil demand, Industria always steals a significant amount of the Hotelling rent even for low-intensity climate damages. In fact, the import tariff component tends to infinity in the long run as the scarcity rents for the marginal unit of oil are infinite. Thus, the import tariff benefits of the carbon tax dominate the Pigouvian global warming benefits in the long run with iso-elastic oil demand.

The implications for Industria’s welfare, however, are ambiguous. As can be seen from Table 3, with unit-elastic oil demand Industria’s welfare in the feedback Nash equilibrium is lower than in the open-loop Nash equilibrium in contrast to the case of linear and semi-loglinear oil demand. The intuition is that the unit-elastic oil demand curve does not set any upper bound on the reservation price. Thus, as Oilrabia and Industria compete to maximize their rent, they set the price too high and end up harming Industria’s consumers, thereby decreasing Industria’s welfare. This implies that the use of the carbon tax to steal rents from the importer is not as beneficial as has hitherto been suggested in the literature as for certain demand specifications it can harm the consumers in the oil-importing nation.

### Non-HARA Production Function: Shifted Unit-Elastic Oil Demand

**Fig. 7:**
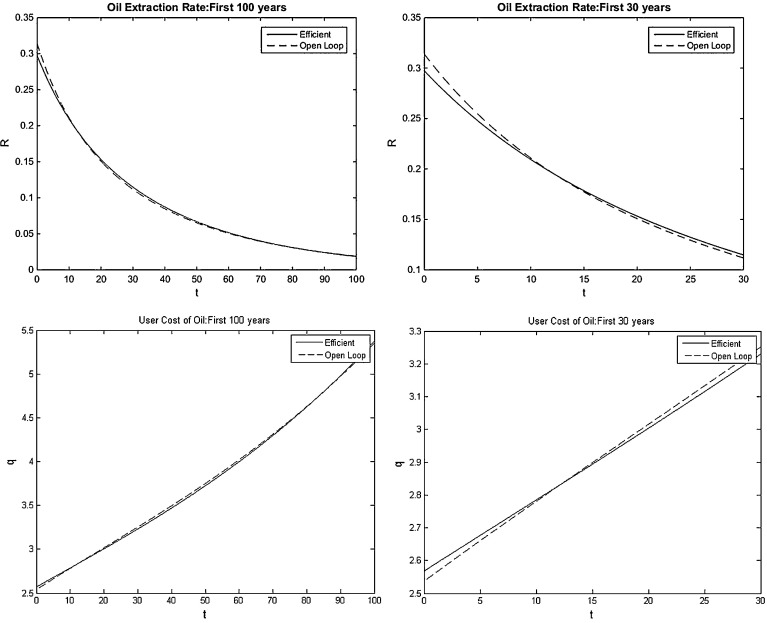
Simulations of oil extraction rate and user cost of oil with non-HARA oil demand

We now examine the non-HARA production function discussed in Sect. 3.5.3 with shifted semi-loglinear oil demand and a negative super-elasticity to investigate whether it is possible for the bias to go in the other direction: the oil extraction rate being initially higher for the open-loop Nash than for the efficient equilibrium. Recall that we postulated in Sect. 3.5.3 that $\chi$ must be small enough to satisfy the no-arbitrage condition (21) but large enough to make this demand specification significantly different from the iso-elastic case. We have numerically solved the open-loop Nash and efficient equilibrium for a range of parameter values with $\phi >1$ and $\chi <\phi (G(S_0 )+{D}'(E_0 )/\rho )$ to find values for which the initial rate of oil extraction is faster for the open-loop Nash equilibrium than for the efficient outcome. One example of such parameter values is $\phi =2.7$ and $\chi =1$. This parameterization leads to: $q_\mathrm{EFF} (0)=2.571$ and $q_\mathrm{OL} (0)=2.558$. Note that the initial user cost of oil in the open-loop Nash equilibrium is lower than in the efficient outcome. The simulation paths for the user cost of oil for the first 100 and the first 30 years for both equilibria are shown in Fig. 7, respectively. We present a separate plot for the initial extraction period as this is when the monopolist behaves differently than with HARA demand.

As Figs. 1 and 5 indicate, with HARA oil demand, the open-loop Nash equilibrium path crosses the efficient path only once. With the shifted semi-loglinear demand example of non-HARA demand, we see that from the second panel of Fig. 7 that there are multiple crossing points. The open-loop Nash equilibrium path starts out steeper than the efficient one and then flattens out, crossing the efficient path again. Thus, initially, the open-loop Nash equilibrium price is lower than the efficient price. This result may seem counterintuitive: Why would the oil price in open-loop Nash equilibrium be lower than in the competitive market outcome? The intuition lies in the behavior of the price elasticity. Imagine that Industria’s population consists of two groups. The first has a fairly low reservation price for oil and can substitute away easily, while the second has a higher reservation price and cannot substitute away from oil. For example, Industria might consist of city dwellers that can switch to public transport and country dwellers that have to drive to work. Thus, for lower oil prices, demand is fairly elastic (city dwellers can stop using oil and switch to public transport) but beyond a certain threshold, the elasticity of demand decreases. The monopolist then initially sets the price slightly lower to capture some of the elastic demand (while the extraction cost is low), and then once all the cheap oil is extracted, it increases the price to serve exclusively the more inelastic portion of demand. As a result of these strategic dynamics, more oil is extracted initially than in the efficient case. Ergo, in the short run, the monopolistic power of Oilrabia is bad for the environment. In the long run, however, the open-loop Nash equilibrium user cost of oil path is flatter than the efficient one just as is the case with HARA preferences.

## Concluding Remarks

Curbing fossil fuel use is essential to prevent further global warming. This goal is complicated by the fact that reserves of fossil fuels are highly concentrated in a few non-western oil-and gas-rich nations with considerable monopolistic power. Previous studies [14, 17, 19, 20, 24] have argued that western oil importers might nevertheless successfully limit fossil fuel consumption via a carbon tax. However, monopolistic exporters are likely to respond to the carbon tax by increasing the oil price more than they would have done in a competitive oil market, which would result in even fewer emissions. In this way, oil exporters can try to capture some of the climate rent. Additionally, the carbon tax can be used as an import tariff to extract scarcity rents from monopolistic oil exporters. This might further benefit the oil-importing nation, but the ability of the importer to capture the monopolist’s scarcity rents depends on the intensity of carbon damages. However, the aforementioned studies limit their attention to linear oil demand in order to keep the analysis tractable. The nature of demand for oil can of course have an important effect on how consumers respond to an increase in the carbon tax. Our paper’s aim was to examine how the specific nature of oil demand affects the oil importer’s efforts to prevent climate change and capture the monopolist’s rents.

We analyze the problem in the framework of a dynamic game between an oil-exporting country with monopoly power on global oil markets and an oil-importing country which is concerned with combating climate change. We find equilibrium conditions for a variety of production functions and their corresponding demand specifications. We prove that for all HARA class production functions, the open-loop Nash equilibrium extraction will initially be slower and later on faster than the efficient rate. As most commonly used demand specifications fall under the HARA class, this implies that in most cases, the monopolist is the conservationist’s best friend. However, we also find a numerical example of a shifted loglinear demand function for which the open-loop equilibrium extraction rate is initially too high. Thus, there are some non-HARA production functions for which in the open-loop equilibrium the oil-exporting country hinders the oil-importing country’s effort to battle climate change. We also solve the model for the feedback Nash equilibrium. Although we do not get any general analytical results, our numerical illustrations indicate that the initial feedback equilibrium consumer price is always higher than in the open-loop Nash equilibrium, which leads to a delay in oil extraction and carbon emissions, and hence lower damages from global warming. This initial consumer price increase is caused by the government of the oil-importing country using the carbon tax as a tariff to steal the oil exporter’s scarcity rents on oil, while the oil-exporting country responds by raising the oil price to steal back some of the climate rents of the oil-importing country. For the iso-elastic production function, the resulting increase in the consumer price of oil leads to a significant welfare loss which outweighs the gain of the captured scarcity rent.

Our main conclusion is that the demand structure plays a significant role when determining the optimal carbon tax or import tariff for foreign oil. Given the number of papers which choose their demand function based on computational convenience, these results serve as a cautionary tale: Demand specifications chosen out of simplicity rather than reflecting reality may make a carbon tax seem more or less beneficial in the context of strategic interactions in regard to exhaustible resources.

While this study generalizes the results of previous studies by examining a range of demand specifications, it remains limited in its scope. For instance, the model we examine excludes the possibility of saving and capital accumulation and a less-stylized carbon cycle dynamics. Without the capital stock, the economy collapses after oil runs out, a highly unrealistic outcome. Meanwhile, without a more sophisticated model of the carbon cycle, all $\hbox {CO}_2$ that is emitted into the atmosphere stays there forever. Thus, we end up over-estimating the amount of climate change. To remedy these problems, we would have to construct a model with additional state variables (for example, using the two reservoirs that [7] uses to model the carbon cycle). The player’s strategies would then be based on the full state space, the two $\hbox {CO}_2$ reservoirs and the oil stock. Finding the feedback equilibrium in this more complex setup would not only make our conclusions more realistic but also provide a contribution to the dynamic game literature through solving for a subgame-perfect Nash equilibrium with multiple state variables, one of which represents a fully exhaustible resource. Another interesting extension would be to introduce multiple oil exporters into the model, similar to [1, 2, 8, 9]. Adding a fringe in addition to the cartel exporter is a better model of reality and would lead to interesting dynamics with the cartel and the fringe responding differently to the importer’s carbon tax. We leave these extensions of our work for further research.

## Appendix 1: Calibration Details

The [11] long-term cost curve for oil puts oil reserves at around 10,000 billion barrels, with initial extraction cost between $5 and $30 per barrel and the highest cost reserves at $115 per barrel. A barrel of oil contains 5.80 mmbtu, and each mmbtu is equal to 20.31 kg $$\hbox {CO}_2$$ [6], so a barrel of oil is 115 kg of carbon or around 1/10 ton of carbon (TC). All of our emission variables are expressed in 1000 Gigaton Carbon (GtC), and prices/costs are expressed in $1000 USD/TC. Initial $$\hbox {CO}_2$$ concentration is set to approximately 800 GtC [6].

We then calibrate the production functions by solving the model for a variety of parameters and checking so that they correspond to real-world data. We calibrate the CARA and quadratic utility production functions so that for zero damages, initial prices and emissions are equal to their 2005 levels. We calibrate the iso-elastic production function so that initial prices and emissions are equal to their 2010 levels (demand amounts are re-scaled to adjust for the population growth between 2005 and 2010). We set the interest rate and the rate of time preference at 1 % per annum.

## Appendix 2: Solving for Equilibria Numerically

The dynamic equilibrium conditions are described by differential equation (13) for the open-loop Nash equilibrium and (27) for the feedback Nash equilibrium. Because we have only one state variable, both problems collapse into a set of differential equations with respect to time, so we can use the same numerical method for both equilibria. We use a Runge–Kutta shooting algorithm, simulating the open-loop Nash and feedback equilibrium user cost of oil using (13) and (31), respectively, and (5) to simulate the path of the oil stock. We do not know the initial value for the user cost of oil $q$, so to find it we introduce a long-run steady-state condition which must hold as time approaches infinity.

For the quadratic and exponential production function, we assume that the marginal productivity at zero is bounded at a low enough value such that some oil is left in situ. Thus, the long-run steady-state condition for the oil stock is:

32 $$\begin{aligned} G(S)+D(E_0 +S_0 -S)/\rho =F^{\prime }(0) \end{aligned}$$

For the iso-elastic and non-HARA production case where the marginal productivity of oil is infinite, the final condition becomes more complex. We describe how to derive the final condition in “Solving for Equilibria with Infinite Marginal Production at Zero” section below). Since the long-run steady state described in (28) is only approached asymptotically and never actually reached, we approximate it by the conditions:

32′ $$\begin{aligned} R<\varepsilon ,\quad G(S)+D(E_0 +S_0 -S)/\rho <\varepsilon \end{aligned}$$

for an arbitrary small value of $\varepsilon$. We use a binary search algorithm to find initial price $q(0)$, which produces a extraction path that satisfies (32). We then use (9) or (29) to back out the open-loop Nash or feedback Nash equilibrium carbon tax, respectively.

### Solving for Equilibria with Infinite Marginal Production at Zero

If the productivity of the marginal unit of oil is greater than the extraction cost of the marginal unit of oil, asymptotically oil is fully exhausted albeit that we never actually get to a point where $R=0,S=0$. Thus, we must find a way to derive a different final condition. Let oil demand growth be $g(t)=\frac{\dot{R}(t)}{R(t)}$. Note that as the oil price increases, oil demand decreases so demand growth is negative. Now assume that as time goes to infinity, the demand growth rate converges to some finite value $\lim _{t\rightarrow \infty } g(t)=\bar{{g}}$ (while we do not have a proof of this conjecture, we can show that it holds numerically). Let $T$ be the time such that $\dot{g}=0$ i.e., $g(T)=\bar{{g}}$. Then, it must hold that for all $t>T$

33 $$\begin{aligned} R(t)=e^{\bar{{g}}(t-T)}R(T). \end{aligned}$$

Substituting (29) in the differential equation for the oil stock (5), we get the condition for the stock of oil at time $T$:

34 $$\begin{aligned} S(T)=R(T)\mathop \int \nolimits _{t=T}^\infty e^{\bar{{g}}(t-T)}dt=-\frac{R(T)}{\bar{{g}}}. \end{aligned}$$

We can now use the same Runge–Kutta algorithm that we used for the quadratic and exponential production function. We find time $T$ numerically by using the Runge–Kutta method to shoot until the growth rate of demand converges and use (30) as the final condition to check whether the oil extraction path satisfies the equilibrium conditions.

## Footnotes

1 van Wijnbergen [23] and van der Meijden et al. [22] analyze two-country general equilibrium models with capital accumulation and international capital flows, but do not look at the strategic interactions of policies in a differential game framework. Jaakkola [12] does do this, but focuses at whether taxation of foreign interest income on oil exporters’ assets helps to overcome the Green Paradox.

2 Wirl [25] demonstrates that decreasing marginal elasticity of utility can boost initial emissions in an international pollution control game, but this study has no exhaustible resource, and both players are affected by climate change.

3 In most cases, studies restrict their attention to interior solutions to make them tractable, so we provide a numerical algorithm which can be used to solve such problems.

4 Groot et al. [8] show that the open-loop Stackelberg equilibrium outcome may give rise to discontinuities in the equilibrium price trajectory. Groot et al. [9] derive the feedback Stackelberg equilibrium outcome for the cartel–fringe model.

5 While utilitarian preferences may imply a variety of utility functions with different elasticities of intertemporal substitution, for computational simplicity, we assume linear utility functions.

6 The social welfare function is linear in the individual utilities.

7 Other informational assumptions are possible (e.g., Industria does not have knowledge of Oilrabia’s stock of remaining oil reserves), but we will abstract from these here.
